# Results of A Study on The Stress Level of Nurses

**DOI:** 10.1192/j.eurpsy.2026.11855

**Published:** 2026-07-31

**Authors:** P. Batchuluun, T. Gantogoo, D. Tserendondog, A. Batsaikhan, M. Dashdondog

**Affiliations:** 1 Department of Medicine Etugen University; 2 Nursing School, Etugen University; 3 Traumatology department, Bayangol District General Hospital, Ulaanbaatar, Mongolia

## Abstract

**Introduction:**

Health professionals, including nurses, are considered a vulnerable group of workers who are susceptible to occupational stress.3 Work stress is the interaction between the work environment and the individual that alters the psychological and physical state of an individual and impairs their ability to function normally. Various work-related stressors, such as heavy workloads and night shifts, contribute to this situation. Therefore, workplace stress and mental health conditions have a negative impact on nurses and their performance. A nurse of high quality of health work force is responsible for high quality and safe care. Palestinian nurse in Palestinians in Palestinians and economic challenge. A high stress level increases the possibility of affecting the effects of a patient’s result.8 Very important to understand the effects of their matters. Therefore, it is necessary to study the stress level of nurses.

**Objectives:**

To study the stress level of nurses

**Methods:**

The study was conducted using the SRQ20 stress test developed by the World Health Organization. A total of 50 nurses participated in the study.

**Results:**

Out of a total of 50 nurses who participated in the study, 30% were 20-24 years old, 40% were 25-34 years old, 16% were 35-39 years old, and 14% were 40-44 years old. Considering the gender ratio of the study participants, 6% were male nurses and 94% were female nurses. Out of a total of 50 nurses who participated in the study, 42% had low stress levels, 42% had medium stress levels, and 16% had high stress levels. When comparing the stress levels of nurses with the departments they work in, 40% of all nurses in the trauma department had high stress levels, 60% had moderate stress levels, and 69.2% of all nurses in the surgical department had moderate stress levels, and 30.8% had low stress levels.

**Conclusions:**

The results of the study show that nurses overall have high levels of workplace stress.

**Disclosure of Interest:**

None Declared

